# Elevated serum sortilin reflects inflammatory burden and coagulation dysfunction in ulcerative colitis

**DOI:** 10.3389/fmed.2026.1811613

**Published:** 2026-04-21

**Authors:** Jiangtao Ding, Haizhou Xu

**Affiliations:** 1 Department of Gastroenterology, Nantong Second People’s Hospital, Chongchuan District, Nantong, Jiangsu, China; 2 Department of General Surgery, Nantong Second People’s Hospital, Chongchuan District, Nantong, Jiangsu, China

**Keywords:** biomarker, coagulation, cytokines, inflammation, sortilin, ulcerative colitis

## Abstract

**Background:**

Sortilin, a multifunctional receptor, is implicated in inflammatory signaling; however, its clinical significance in ulcerative colitis (UC) remains unclear. This study evaluated serum sortilin levels in patients with UC and their associations with disease activity, inflammation, and coagulation.

**Methods:**

Serum sortilin, inflammatory cytokines (TNF-α, IL-1β, IL-6, IL-17A, and IL-10), and coagulation indices (fibrinogen and D-dimer) were measured in patients with UC and healthy controls. Patients were stratified according to disease activity and severity. Correlations between serum sortilin levels and clinical, biochemical, and inflammatory parameters were assessed. Receiver operating characteristic (ROC) curve analysis was performed to evaluate the diagnostic value of serum sortilin.

**Results:**

Serum sortilin levels were significantly higher in patients with UC than in healthy controls (*p* < 0.001) and were further elevated in active UC compared to those in remission (*p* < 0.001). Sortilin concentrations increased progressively with disease severity. Positive correlations were observed between serum sortilin and CRP (*r* = 0.522), TNF-α (*r* = 0.576), IL-1β (*r* = 0.442), IL-6 (*r* = 0.684), IL-17A (*r* = 0.492), fibrinogen (*r* = 0.267), D-dimer (*r* = 0.543), platelet count (*r* = 0.257), white blood cell count (*r* = 0.214), and Mayo score (*r* = 0.615) (all *p* < 0.05). In contrast, negative correlations were found with IL-10 (*r* = −0.609) and hemoglobin (*r* = −0.226; both *p* < 0.05), whereas no significant association was observed with albumin (*p* = 0.584). ROC analysis demonstrated high diagnostic accuracy for serum sortilin in distinguishing UC patients from controls [area under the curve (AUC) = 0.944; sensitivity, 83.2%; specificity, 93.8%] and in differentiating severe from mild UC (AUC = 0.968; sensitivity, 86.4%; specificity, 96.3%).

**Conclusion:**

Elevated serum sortilin levels are closely associated with disease activity, inflammatory cytokines, and coagulation parameters in UC, suggesting that sortilin may serve as a novel biomarker reflecting both inflammatory and hypercoagulable states in UC pathogenesis.

## Introduction

Ulcerative colitis (UC) is a chronic idiopathic inflammatory bowel disease characterized by a relapsing-remitting course of mucosal inflammation, primarily involving the colon and rectum (1). This inflammation is frequently accompanied by systemic manifestations, including immune activation, a pro-coagulant state, and extraintestinal complications (2–4). An accurate assessment of disease severity and biomarkers that reflect the interplay between inflammation and coagulation are critically important for guiding therapy and monitoring outcomes in UC.

From an inflammatory perspective, elevated systemic markers, such as C-reactive protein (CRP), erythrocyte sedimentation rate (ESR), neutrophil-to-lymphocyte ratio (NLR), and platelet-to-lymphocyte ratio (PLR), have been associated with more active disease and more extensive colonic involvement in UC (1, 2, 5). Although broadly useful, these markers are nonspecific and may not fully capture mucosal or submucosal changes. Thus, ongoing research is seeking novel biomarkers that better reflect disease activity, severity, and prognosis (1).

In parallel with the inflammatory response, there is growing recognition that UC is associated with alterations in coagulation and fibrinolysis. Samples from patients with active disease show increased fibrinogen levels, elevated D-dimer and fibrin/fibrinogen degradation products (FDP), activated platelets, and shortened or altered prothrombin/activated partial thromboplastin times, all of which suggest a hypercoagulable state (2, 5, 6). Moreover, such coagulation changes correlate with markers of inflammation and may reflect a mechanistic link between mucosal injury, systemic inflammation, and thrombotic risk.

Sortilin is emerging as a multifunctional regulator of cellular trafficking, lipid metabolism, and immune and inflammatory pathways (7–9). Sortilin functions as a high-affinity receptor for pro-inflammatory cytokines such as interleukin-6 (IL-6) and interferon-γ (IFN-γ); its genetic deletion in immune cells reduces cytokine secretion and atherogenesis in experimental models, highlighting its role in modulating inflammation (7). Additionally, circulating sortilin levels have been associated with cardiovascular disease severity and adverse outcomes, further linking them to systemic vascular and inflammatory processes (2, 5, 10). While the majority of research has focused on cardiovascular and metabolic disorders, the potential relevance of sortilin in inflammatory and coagulation-linked diseases beyond these domains is increasingly recognized.

Given the confluence of inflammation, coagulation dysregulation, and mucosal injury in UC, investigating whether serum sortilin reflects these pathobiological axes is of particular interest. Specifically, it has been hypothesized that elevated serum sortilin levels may be associated with greater disease severity, more pronounced coagulation activation, and heightened systemic inflammation in UC. However, this investigation aimed to evaluate the relationships between serum sortilin levels, UC severity, coagulation/fibrinolysis markers, and established inflammation indices. Thus, we sought to determine whether sortilin may serve as a novel biomarker linking mucosal inflammation to systemic coagulation activation in UC and whether it may help stratify disease severity or predict complications.

## Patients and methods

### Study population

This retrospective study included 63 patients with UC in remission and 92 patients with active UC who were treated at the Department of Gastroenterology of our hospital between January 2021 and June 2025. The diagnosis of UC was established based on a combination of clinical manifestations and laboratory, imaging, endoscopic, and histopathologic findings (11, 12) in accordance with established diagnostic criteria. Eligible participants were adults aged ≥ 18 years with a first diagnosis of UC and complete clinical data available.

To minimize potential confounding effects of pharmacological therapy on inflammatory cytokines and coagulation parameters, treatment status at the time of blood sampling was carefully reviewed. Patients in the active UC group were newly diagnosed or had not received systemic corticosteroids, immunomodulators, or biologic agents for at least 4 weeks prior to sample collection. Patients in the remission group were receiving stable maintenance therapy, primarily 5-aminosalicylic acid preparations, without recent treatment escalation. None of the enrolled patients were receiving anticoagulant therapy at the time of blood sampling.

Patients were excluded if they had concomitant autoimmune or systemic inflammatory diseases, acute or chronic infections, Crohn’s disease, prior colorectal surgery, hepatic or renal dysfunction, coagulation disorders, malignancy, or recent use of immunosuppressive or anticoagulant medications. These criteria were applied to ensure that the observed biochemical, inflammatory, and coagulation changes were attributable specifically to UC.

**FIGURE 1:**
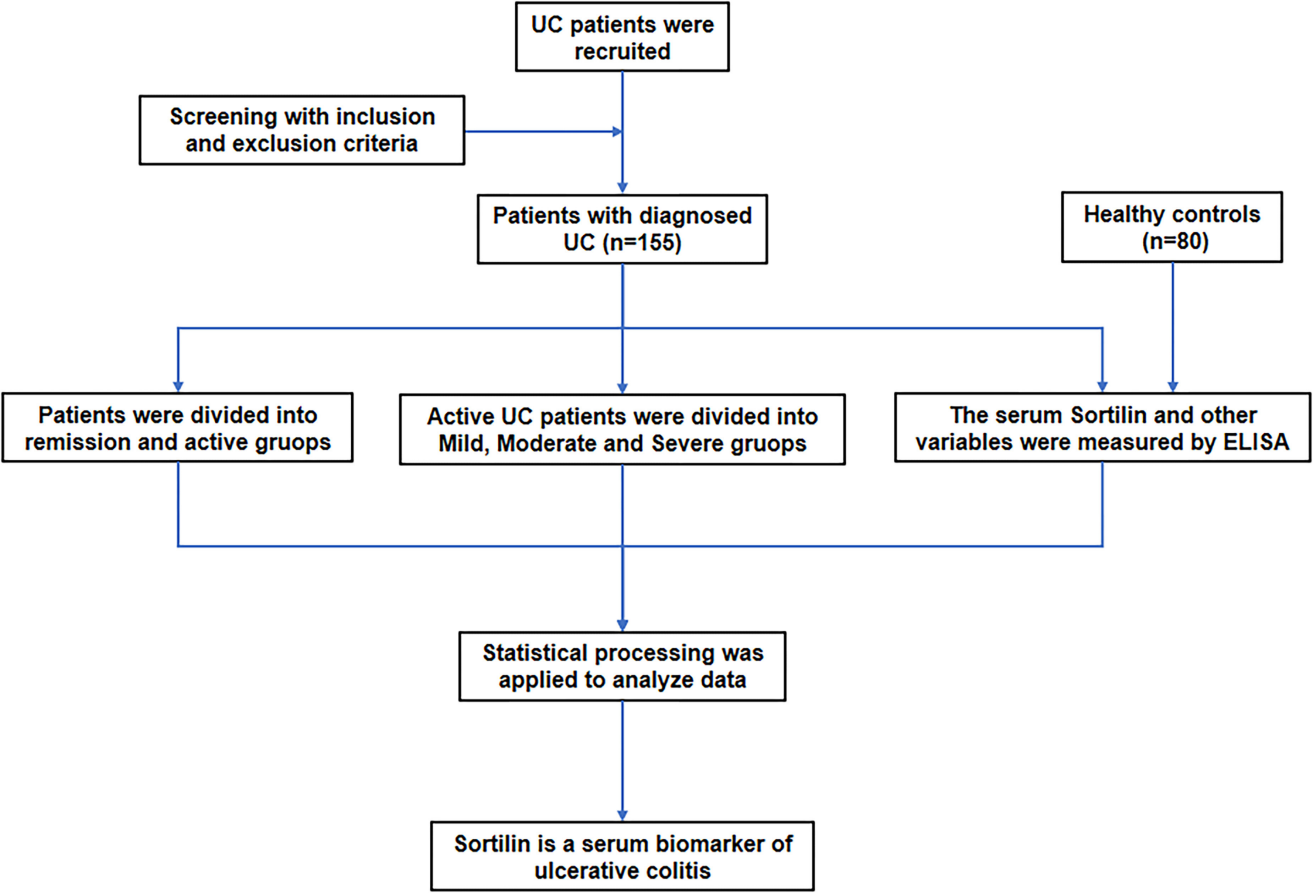
Schematic overview of the study workflow and analytical framework. The diagram illustrates the overall study design, with arrows indicating the sequential flow of participant grouping, data collection and downstream analyses. Horizontal connections represent parallel comparisons, and vertical arrows denote progression through the study stages and outcome assessments.

The control group consisted of 80 age- and sex-matched healthy individuals undergoing routine physical examinations at our hospital. The clinical research workflow, measurement indicators, and statistical analyses are summarized in Figure 1. The study protocol was reviewed and approved by the Ethics Committee of our hospital, and all procedures were conducted in accordance with the Declaration of Helsinki.

### Severity assessment of disease

Disease activity in patients with ulcerative colitis was assessed using a standardized endoscopic disease activity scoring system. Endoscopic findings were graded as follows: remission (score = 0), mild disease (score = 1), moderate disease (score = 2), and severe disease (score = 3), as previously described (13). Based on this classification, patients were categorized into remission or active disease groups, with the active disease group further stratified into mild, moderate, and severe subgroups for subsequent analyses.

### Data collection

Demographic and clinical data, including age, sex, and body mass index (BMI), were collected from each patient. Venous blood was obtained and measured within 24 h of admission (fasting). Serum albumin, hemoglobin, white blood cell (WBC) count, platelet count, erythrocyte sedimentation rate (ESR), and C-reactive protein (CRP) levels were measured using an automatic biochemical analyzer. D-Dimer and fibrinogen were measured using automated coagulation analyzer.

### Enzyme-Linked Immunosorbent Assay

Fasting venous blood samples were collected from all participants and centrifuged to obtain serum. The separated serum samples were aliquoted and stored at −80°C until analysis. Serum concentrations of Tumor Necrosis Factor alpha (TNF-α; DTA00D, R&D Systems), Interleukin-1 beta (IL-1β; DLB50, R&D Systems), Interleukin-6 (IL-6; D6050B, R&D Systems), Interleukin-10 (IL-10; D1000B, R&D Systems), Interleukin-17A (IL-17A; D1700, R&D Systems), and Sortilin (CSB-EL022412HU, CUSABIO, China) were quantified using commercially available ELISA kits, according to the manufacturer’s instructions. All measurements were performed in duplicates to ensure assay reliability. According to the manufacturer’s specifications, the intra-assay and inter-assay coefficients of variation for all assays were < 10% and < 12%, respectively, indicating acceptable assay precision and reproducibility. Optical density values were measured using a microplate reader, and the concentrations were calculated based on standard curves generated for each biomarker.

### Statistical analysis

Statistical analyses were performed using IBM SPSS Statistics version 20.0 (IBM). Continuous variables were assessed for normality using the Shapiro-Wilk test and are presented as mean ± standard deviation when normally distributed. Group comparisons were conducted using the independent samples *t*-test or one-way analysis of variance (ANOVA), as appropriate. Categorical variables are expressed as frequencies and were compared using the chi-square test. Correlations between serum sortilin levels and clinical, inflammatory, and coagulation parameters were evaluated using Pearson’s correlation for normally distributed variables and Spearman’s rank correlation for non-normally distributed variables. To control for multiple comparisons, the Bonferroni correction was applied to all correlation analyses. Receiver operating characteristic (ROC) curve analysis was used to assess the diagnostic performance of serum sortilin and to determine the optimal cutoff value for distinguishing patients with ulcerative colitis from healthy controls. A two-sided adjusted p-value < 0.05 was considered statistically significant.

## Results

### Demographic and biochemical characteristics

The demographic and biochemical characteristics of patients with UC and healthy controls are summarized in Table 1. Age and sex distributions did not differ significantly among the control, remission UC, and active UC groups. Body mass index was significantly lower in the active UC group than in the remission and control groups (*p* = 0.003). Serum albumin and hemoglobin levels showed a progressive decline from controls to remission UC and active UC (both *p* < 0.001). Markers of systemic inflammation, including white blood cell count, platelet count, erythrocyte sedimentation rate, and C-reactive protein, were significantly higher in patients with active UC than in those with remission UC and controls (all *p* < 0.001). Coagulation parameters, including D-dimer and fibrinogen, increased significantly with disease activity (both *p* < 0.001). Circulating concentrations of TNF-α, IL-1β, IL-6, and IL-17A were significantly elevated in the active UC group, whereas IL-10 levels were significantly reduced with increasing disease activity (all *p* < 0.001). Serum sortilin levels were significantly higher in patients with UC than in healthy controls and were highest in those with active disease (*p* < 0.001).

**TABLE 1:** Clinic and laboratory characteristics of study population.

Variable	Controls (*n* = 80)	Remission UC (*n* = 63)	Active UC (*n* = 92)	*p*-value
Age (years)	42.95 ± 9.66	43.17 ± 9.32	42.68 ± 8.97	0.948
Sex (male, %)	42 (52.5%)	35 (55.6%)	49 (53.3%)	0.932
BMI (kg/m^2^)	24.72 ± 2.83	24.68 ± 2.66	23.45 ± 2.58	0.003
Serum albumin (g/L)	42.61 ± 3.61	39.20 ± 3.74	37.53 ± 3.40	<0.001
Hemoglobin (g/L)	140.49 ± 14.07	132.67 ± 17.10	125.59 ± 15.31	<0.001
WBC (×10^9^/L)	6.10 ± 1.34	7.83 ± 2.48	9.52 ± 3.36	<0.001
Platelet (×10^9^/L)	218.35 ± 50.56	236.80 ± 49.42	268.34 ± 50.09	<0.001
ESR (mm/h)	7.06 ± 1.91	9.70 ± 2.35	17.29 ± 5.49	<0.001
D-Dimer (μg/L)	211.79 ± 32.40	272.26 ± 43.34	537.45 ± 112.81	<0.001
Fibrinogen (g/L)	2.37 ± 0.39	2.64 ± 0.43	3.53 ± 0.83	<0.001
CRP (μg/mL)	2.90 ± 0.44	8.86 ± 1.37	17.86 ± 4.43	<0.001
TNF-α (pg/mL)	47.66 ± 6.39	58.01 ± 6.52	90.23 ± 18.51	<0.001
IL-1β (pg/mL)	2.22 ± 0.39	2.83 ± 0.41	4.63 ± 0.95	<0.001
IL-6 (pg/mL)	5.45 ± 0.66	9.41 ± 1.20	19.27 ± 5.56	<0.001
IL-10 (pg/mL)	52.81 ± 6.52	47.96 ± 6.21	35.98 ± 6.60	<0.001
IL-17A (pg/mL)	39.15 ± 5.44	49.19 ± 6.30	77.22 ± 15.16	<0.001
Sortilin (pg/mL)	13.86 ± 2.32	18.10 ± 2.83	26.46 ± 5.92	<0.001

*T*-test is applied to compare the differences between two groups. BMI, body mass index; WBC, white blood cells; ESR, erythrocyte sedimentation rate; CRP, C-reactive protein; TNF-α, tumor necrosis factor-α; IL-1β, interleukin 1β; IL-6, interleukin 6; IL-10, interleukin 10; IL-17A, interleukin 17A.

### Clinical and laboratory characteristics according to UC severity

The clinical and laboratory characteristics of patients with UC stratified by disease severity are summarized in Table 2. No significant differences were observed among the mild, moderate, and severe UC groups with respect to age, sex, body mass index, serum albumin, or hemoglobin levels (all *p* > 0.05). Inflammatory parameters increased significantly with disease severity. White blood cell counts were higher in patients with severe UC than in those with mild and moderate disease (*P* < 0.001). Erythrocyte sedimentation rate and C-reactive protein levels also increased progressively across severity categories (both *p* < 0.001). Coagulation-related markers demonstrated a similar trend. D-dimer and fibrinogen levels were significantly elevated in patients with severe UC compared with those with mild and moderate disease (both *p* < 0.001). Pro-inflammatory cytokines, including TNF-α, IL-1β, IL-6, and IL-17A, increased significantly with increasing disease severity, whereas IL-10 levels decreased progressively (all *p* < 0.001). Serum sortilin concentrations increased stepwise from mild to moderate and severe UC (*p* < 0.001).

**TABLE 2:** Clinic and laboratory characteristics of colitis patients with different severity.

Variable	Mild group (*n* = 27)	Moderate group (*n* = 43)	Severe group (*n* = 22)	*p*-value
Age (years)	42.56 ± 9.29	43.09 ± 9.09	42.05 ± 8.72	0.904
Sex (male, %)	15 (55.6%)	22 (51.2%)	12 (54.5%)	0.929
BMI (kg/m^2^)	23.64 ± 2.44	23.67 ± 2.65	22.77 ± 2.63	0.373
Serum albumin (g/L)	38.49 ± 3.54	37.47 ± 3.34	36.49 ± 3.14	0.123
Hemoglobin (g/L)	129.19 ± 16.59	125.70 ± 14.75	120.95 ± 14.16	0.068
WBC (×10^9^/L)	8.34 ± 2.57	9.08 ± 2.93	11.81 ± 3.98	<0.001
Platelet (×10^9^/L)	251.27 ± 46.68	271.22 ± 48.48	283.68 ± 53.26	0.180
ESR (mm/h)	12.12 ± 2.80	17.65 ± 4.16	22.94 ± 4.26	<0.001
D-Dimer (μg/L)	427.46 ± 62.73	542.99 ± 77.80	661.59 ± 79.67	<0.001
Fibrinogen (g/L)	3.15 ± 0.70	3.49 ± 0.82	4.06 ± 0.74	<0.001
CRP (μg/mL)	14.03 ± 2.13	17.47 ± 3.08	23.31 ± 3.23	<0.001
TNF-α (pg/mL)	74.36 ± 9.21	89.17 ± 13.25	111.78 ± 14.65	<0.001
IL-1β (pg/mL)	3.61 ± 0.56	4.87 ± 0.66	5.43 ± 0.70	<0.001
IL-6 (pg/mL)	13.22 ± 1.65	19.59 ± 3.31	26.09 ± 3.55	<0.001
IL-10 (pg/mL)	42.01 ± 4.94	36.25 ± 4.17	28.05 ± 3.31	<0.001
IL-17A (pg/mL)	62.94 ± 8.08	77.25 ± 10.68	94.70 ± 10.22	<0.001
Sortilin (pg/mL)	21.30 ± 3.52	26.49 ± 4.26	32.73 ± 4.97	<0.001

BMI, body mass index; WBC, white blood cells; ESR, erythrocyte sedimentation rate; CRP, C-reactive protein; TNF-α, tumor necrosis factor-α; IL-1β, interleukin 1β; IL-6, interleukin 6; IL-10, interleukin 10; IL-17A, interleukin 17A.

### Comparison of serum sortilin between healthy controls and patients with UC

**FIGURE 2:**
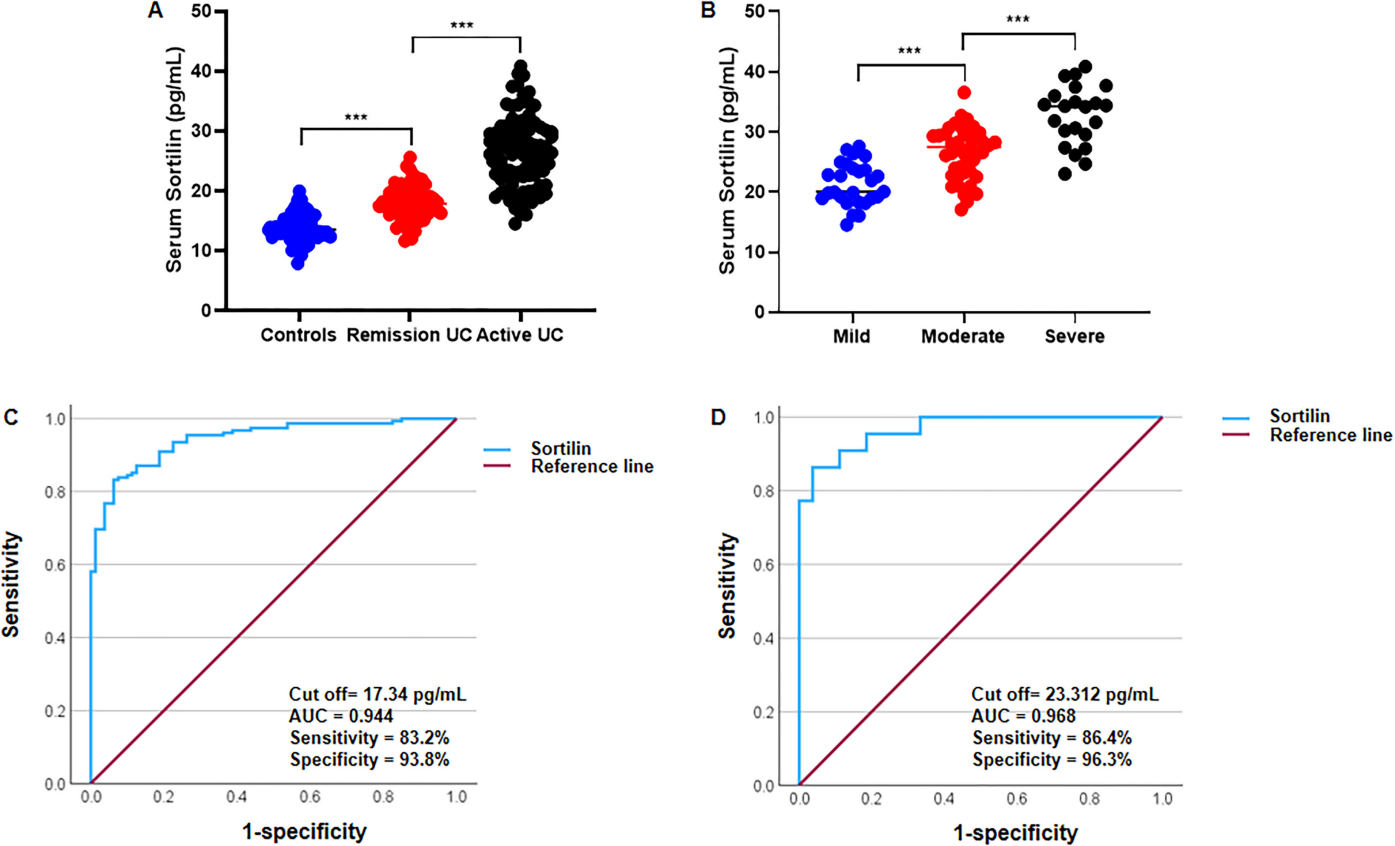
Comparison of serum sortilin in healthy controls and UC patients. **(A)** Serum sortilin levels were compared in control subjects (*n* = 80), remission UC patients (*n* = 63) and active UC patients (*n* = 92). Disease activity was classified according to endoscopic scores as follows: remission (score 0), mild (score 1), moderate (score 2), and severe (score 3). Serum sortilin concentrations were measured by ELISA. **(B)** Serum sortilin levels were compared in active UC patients of mild group (*n* = 27), moderate (*n* = 43) and severe (*n* = 22). **(C)** The ROC curve is used to obtain the critical point of serum sortilin levels that distinguish between healthy controls and UC patients. **(D)** The ROC curve was used to identify the critical serum sortilin level that distinguished between patients with mild and severe UC. The optimal critical point was 23.312 pg/mL. The optimal critical point is 17.34 pg/mL. ANOVA is used to compare the differences between three groups, followed by LSD test for multiple comparisons. UC, ulcerative colitis. ***P < 0.001.

Serum sortilin levels were significantly higher in patients with UC than in healthy controls (*p* < 0.001; Figure 2A). Within the UC cohort, serum sortilin concentrations were also significantly higher in patients with severe UC than in those with mild and moderate disease (*p* < 0.001; Figure 2B). Receiver operating characteristic (ROC) curve analysis showed that serum sortilin differentiated patients with UC from healthy controls with an area under the curve (AUC) of 0.944, a cutoff value of 17.34 pg/mL, sensitivity of 83.2%, and specificity of 93.8% (Figure 2C). ROC analysis further demonstrated discrimination between severe and mild UC, with an AUC of 0.968, a cutoff value of 23.312 pg/mL, sensitivity of 86.4%, and specificity of 96.3% (Figure 2D).

### Diagnostic performance of inflammatory markers and sortilin

**FIGURE 3:**
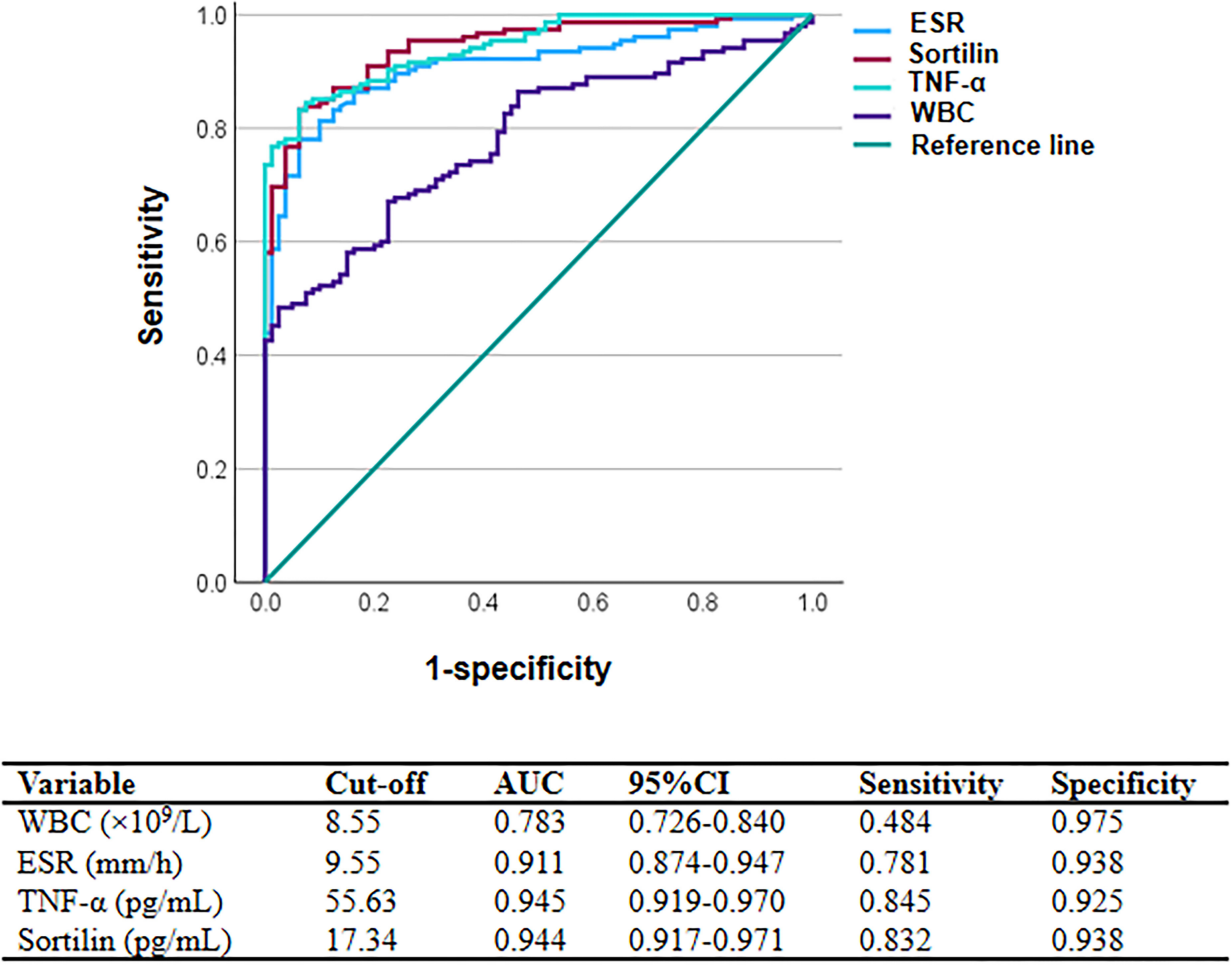
The ROC curve analysis of biomarkers for disease severity in colitis. ROC curves illustrate the diagnostic performance of white blood cell count (WBC), erythrocyte sedimentation rate (ESR), tumor necrosis factor-α (TNF-α), and serum sortilin in discriminating disease severity in patients with colitis. Sensitivity is plotted against 1-specificity. The diagonal line represents the reference line, indicating no discriminative ability.

ROC curve analysis was conducted to assess the discriminatory performance of WBC, ESR, TNF-α, and serum sortilin for disease severity in patients with colitis (Figure 3). ESR showed an AUC of 0.911 (95% CI: 0.874–0.947) with a sensitivity of 0.781 and specificity of 0.938 at a cut-off of 9.55 mm/h. TNF-α demonstrated an AUC of 0.945 (95% CI: 0.919–0.970), with sensitivity and specificity of 0.845 and 0.925, respectively, at a cut-off of 55.63 pg/mL. Serum sortilin yielded an AUC of 0.944 (95% CI: 0.917–0.971), with a sensitivity of 0.832 and specificity of 0.938 at a cut-off of 17.34 pg/mL. WBC showed an AUC of 0.783 (95% CI: 0.726–0.840), with sensitivity of 0.484 and specificity of 0.975 at a cut-off of 8.55 × 10^9^/L.

### Correlation between serum sortilin levels and routine blood test and Mayo score in patients with UC

**FIGURE 4:**
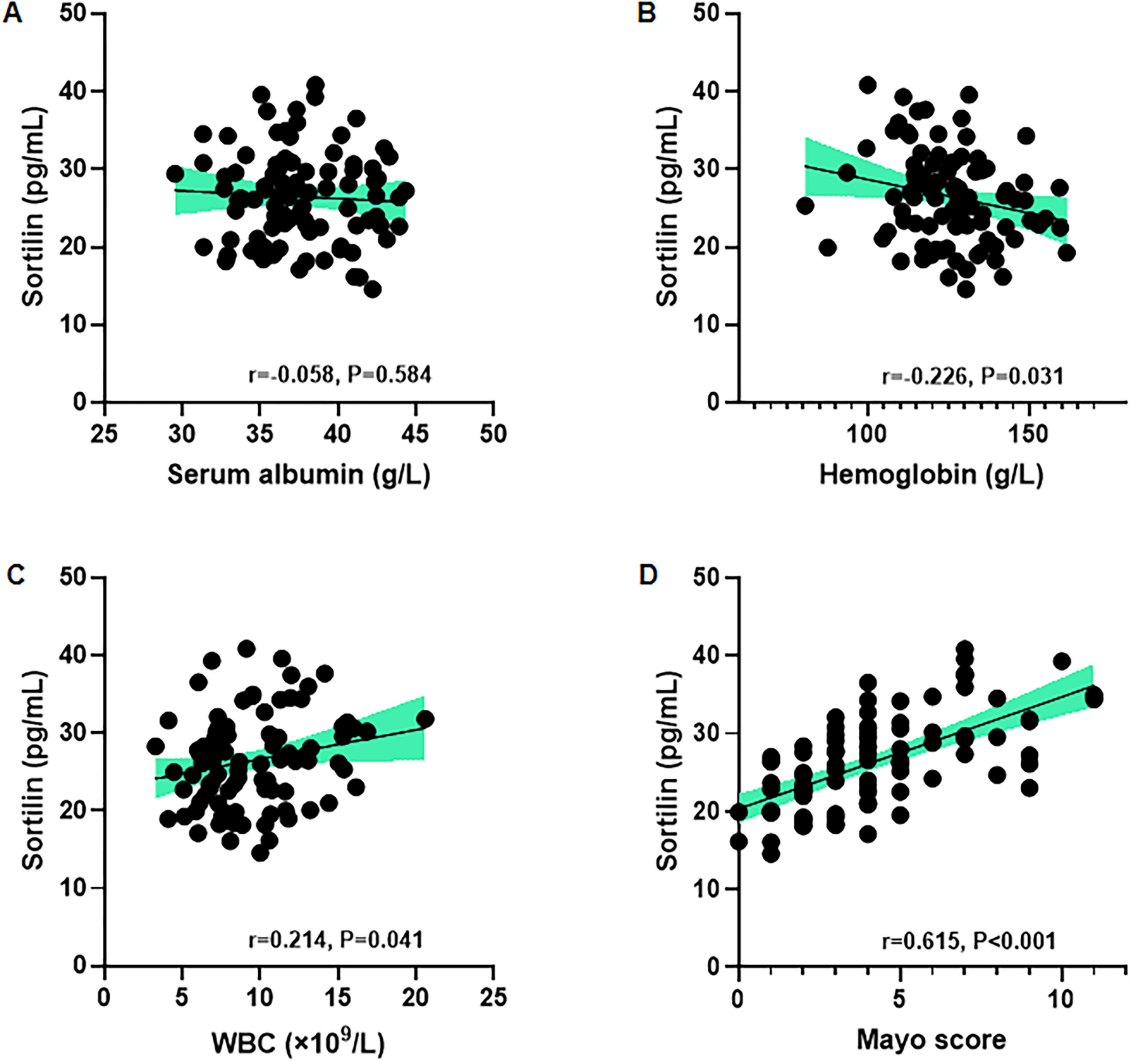
Correlation between serum sortilin levels and routine blood test and Mayo score in UC patients. Serum sortilin is negatively correlated with **(A)** serum albumin, **(B)** hemoglobin, and positively correlated with **(C)** WBC and **(D)** Mayo score. Correlational analyses were performed using Pearson or Spearman correlations, as appropriate, based on the data distribution. ESR, erythrocyte sedimentation rate; WBC, white blood cells.

Correlation analyses revealed that serum sortilin levels were significantly associated with multiple routine blood test results and the Mayo score in patients with UC (Figure 4). No significant correlation was observed between serum sortilin and albumin levels (*r* = −0.058, *p* = 0.584), suggesting that serum sortilin concentrations may not be linked to nutritional status. Serum sortilin showed a strong negative correlation with hemoglobin (*r* = −0.226, *p* = 0.031). Additionally, sortilin levels were positively correlated with WBC (*r* = 0.214, *p* = 0.041) and Mayo score (*r* = 0.615, *p* < 0.001), reflecting its involvement in systemic inflammation and disease severity. Collectively, these findings demonstrate that elevated serum sortilin levels reflect both the inflammatory burden in patients with UC.

### Correlation between serum sortilin levels and coagulation related indicators in patients with UC

**FIGURE 5:**
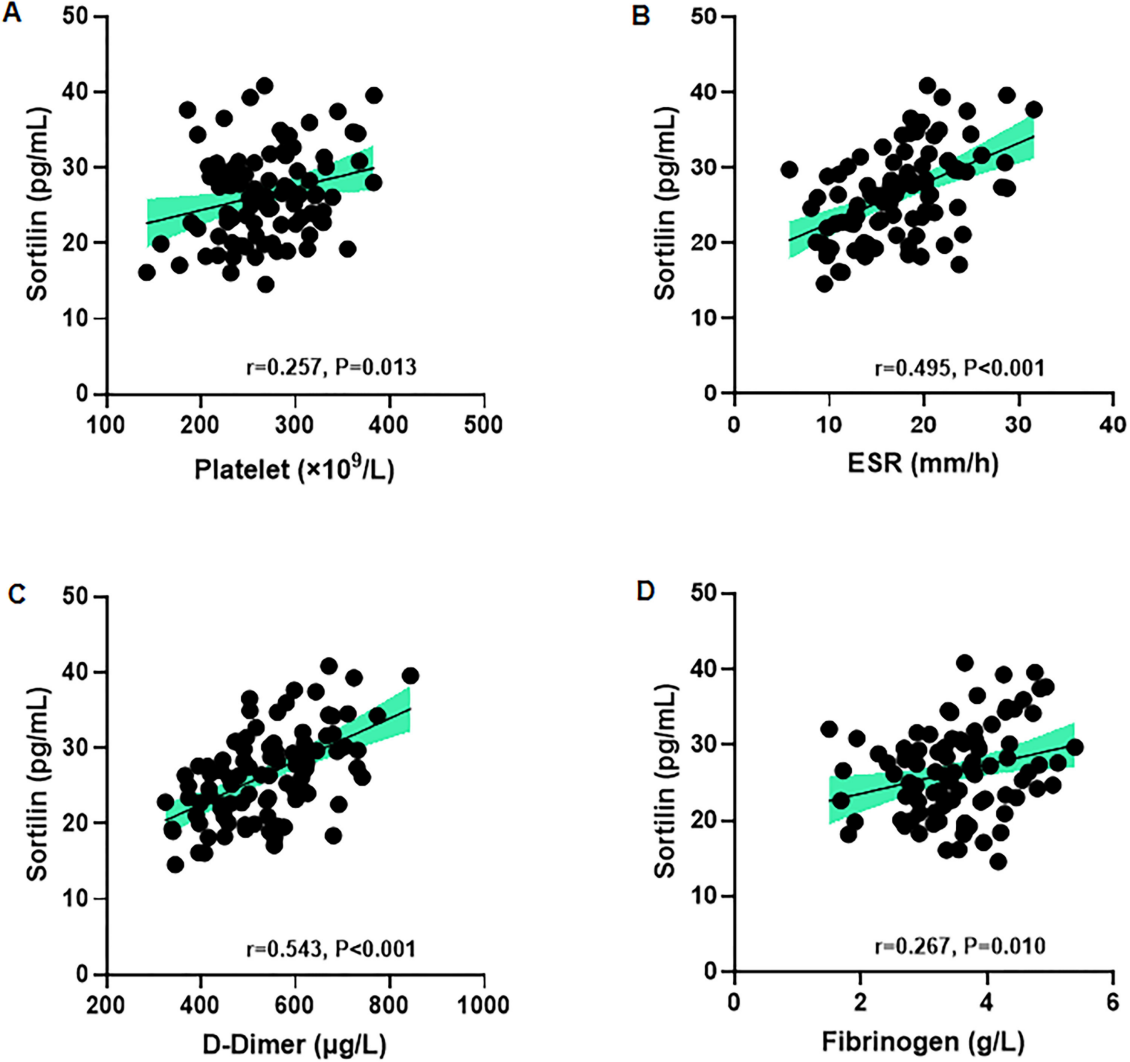
Correlation between serum sortilin levels and coagulation related indicators in UC patients. Serum sortilin is positively correlated with **(A)** platelet count, **(B)** ESR, **(C)** D-Dimer and **(D)** fibrinogen. Correlations were analyzed using Pearson or Spearman correlations according to variable distribution. ESR, erythrocyte sedimentation rate.

Correlation analyses showed that serum sortilin levels were positively associated with several inflammatory and coagulation-related parameters in patients with UC (Figure 5). Significant positive correlations were observed between serum sortilin and platelet count (*r* = 0.257, *p* = 0.013), ESR (*r* = 0.495, *p* < 0.001), D-dimer (*r* = 0.543, *p* < 0.001), and fibrinogen (*r* = 0.267, *p* = 0.010), indicating a close association of serum sortilin levels with enhanced coagulation activity and platelet activation during active disease.

### Correlation between serum sortilin and inflammatory markers in UC

**FIGURE 6:**
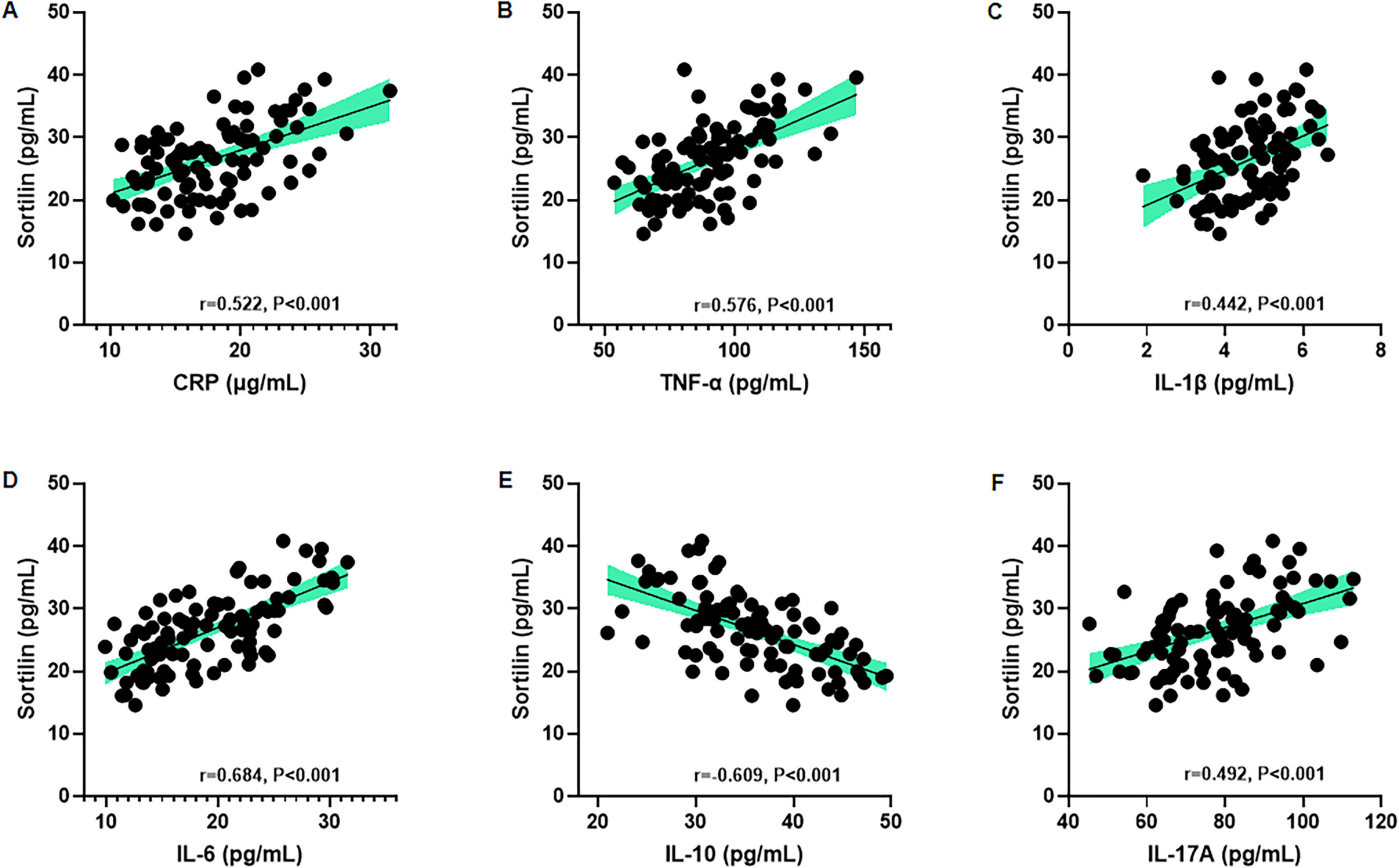
Correlation between serum sortilin levels and inflammatory indicators of UC patients. Serum sortilin is positively correlated with **(A)** CRP, **(B)** TNF-α, **(C)** IL-1β, **(D)** IL-6, negatively correlated with **(E)** IL-10, and positively correlated with **(F)** IL-17A. Correlational analyses were conducted using Pearson or Spearman correlations, as appropriate. CRP, C-reactive protein; TNF-α, tumor necrosis factor-α; IL-1β, interleukin 1β; IL-6, interleukin 6; IL-10, interleukin 10; IL-17A, interleukin 17A.

Correlation analyses demonstrated that serum sortilin levels were positively correlated with CRP (*r* = 0.522, *p* < 0.001), TNF-α (*r* = 0.576, *p* < 0.001), IL-1β (*r* = 0.442, *p* < 0.001), IL-6 (*r* = 0.684, *p* < 0.001), and IL-17A (*r* = 0.492, *p* < 0.001) (Figures 6A–D,F). In contrast, serum sortilin levels were negatively correlated with the anti-inflammatory cytokine IL-10 (*r* = −0.609, *p* < 0.001) (Figure 6E).

## Discussion

In this study, we demonstrated that serum sortilin levels were significantly elevated in patients with UC compared to healthy controls and increased in proportion to endoscopic disease severity. Importantly, serum sortilin levels were strongly correlated with systemic inflammatory markers, including CRP, ESR, WBC count, TNF-α, IL-1β, IL-6, and IL-17A, and inversely associated with the anti-inflammatory cytokine IL-10. Additionally, sortilin levels were positively associated with markers of coagulation activation, such as fibrinogen, D-dimer, and platelet count. ROC analysis further showed that serum sortilin had high diagnostic performance in distinguishing UC patients from healthy individuals, with an AUC comparable to that of established inflammatory biomarkers.

Our findings align with the well-characterized systemic inflammatory phenotype of UC. Active UC is associated with heightened Th17-driven immune responses, reflected by elevated IL-17A, IL-6, and other pro-inflammatory cytokines (14, 15). These cytokine elevations are known to correlate with mucosal damage and clinical severity (16). The strong positive correlations between SORTILIN and CRP, ESR, TNF-α, and IL-6 observed in our cohort suggest that sortilin may reflect the systemic inflammatory burden in UC, complementing traditional serum markers.

Sortilin (encoded by SORT1) is a multi-functional sorting receptor implicated in intracellular trafficking, lipid metabolism, and immune regulation (4, 17). Emerging evidence suggests that sortilin can modulate cytokine secretion pathways and influence innate immune signaling. For example, sortilin facilitates the trafficking and release of pro-inflammatory cytokines, such as IL-6 and IFN-γ, in macrophages and other immune cells, and binds to multiple inflammatory mediators (17, 18). Our study provides clinical evidence supporting these mechanistic insights: as UC severity increased, so did serum sortilin levels, mirroring the rise in pro-inflammatory cytokines and higher endoscopic scores.

The negative correlation between sortilin and IL-10 further highlights its association with a pro-inflammatory milieu. IL-10 is a key anti-inflammatory cytokine that suppresses Th17 responses and promotes mucosal tolerance (19). Reduced IL-10 levels in active UC have been reported previously and correlated with disease activity (20, 21). The inverse association between sortilin and IL-10 in our data suggests that sortilin elevation may be part of a dysregulated immune response characteristic of active UC.

In addition to inflammation, UC is associated with a procoagulant state and thromboembolic risk (22). Chronic mucosal inflammation promotes endothelial activation, platelet aggregation, and upregulation of the coagulation cascade, manifesting as elevated fibrinogen and D-dimer levels (23). The positive correlations between sortilin and fibrinogen, D-dimer, and platelet count in our cohort support an association between sortilin and a hypercoagulable state in UC. This relationship complements preclinical evidence suggesting that sortilin interacts with lipid and coagulation pathways and may influence thrombosis risk (24).

The high diagnostic performance of serum sortilin (AUC = 0.944) in distinguishing UC from healthy controls is notable. Although CRP, ESR, and TNF-α remain widely used biomarkers of UC activity, they lack specificity and may be elevated in other inflammatory conditions (25). Serum sortilin’s specificity of 93.8% in our cohort suggests potential utility as an adjunctive biomarker. However, the lack of comparison with other gastrointestinal and systemic inflammatory diseases in this study limits our conclusions about its diagnostic specificity.

## Limitations

This study has several limitations that should be acknowledged. First, although the sample size was adequate for an exploratory, hypothesis-generating biomarker study, the cross-sectional design permits the identification of associations but does not allow causal inferences. While elevated serum sortilin levels were closely associated with inflammatory and coagulation markers, it remains unclear whether sortilin acts as a driver of inflammation or is a downstream consequence of inflammatory activity. Future mechanistic investigations, including *in vitro* and animal model studies, are warranted to clarify its role in colonic inflammation. Second, this was a single-center study, and larger prospective, multi-center cohorts, including diverse and multi-ethnic populations, are required to validate the diagnostic performance of serum sortilin and confirm the generalizability of our findings. Finally, serial measurements of sortilin were not performed; longitudinal assessments tracking transitions from active disease to remission would provide stronger evidence for its potential utility as a disease-monitoring biomarker.

Despite these limitations, our results suggest that serum sortilin is a promising integrative biomarker that reflects both inflammatory burden and coagulation activation in UC. Its strong associations with disease severity, systemic cytokines, and coagulation indices position it as a candidate for future prospective studies. Longitudinal and multi-center investigations should evaluate whether serial sortilin measurements can reliably track treatment response, predict flare-ups, or stratify thrombotic risk. Additionally, mechanistic exploration in cell and animal models may reveal whether sortilin plays a functional role in mucosal immune regulation or coagulation dynamics in UC.

## Conclusion

**FIGURE 7:**
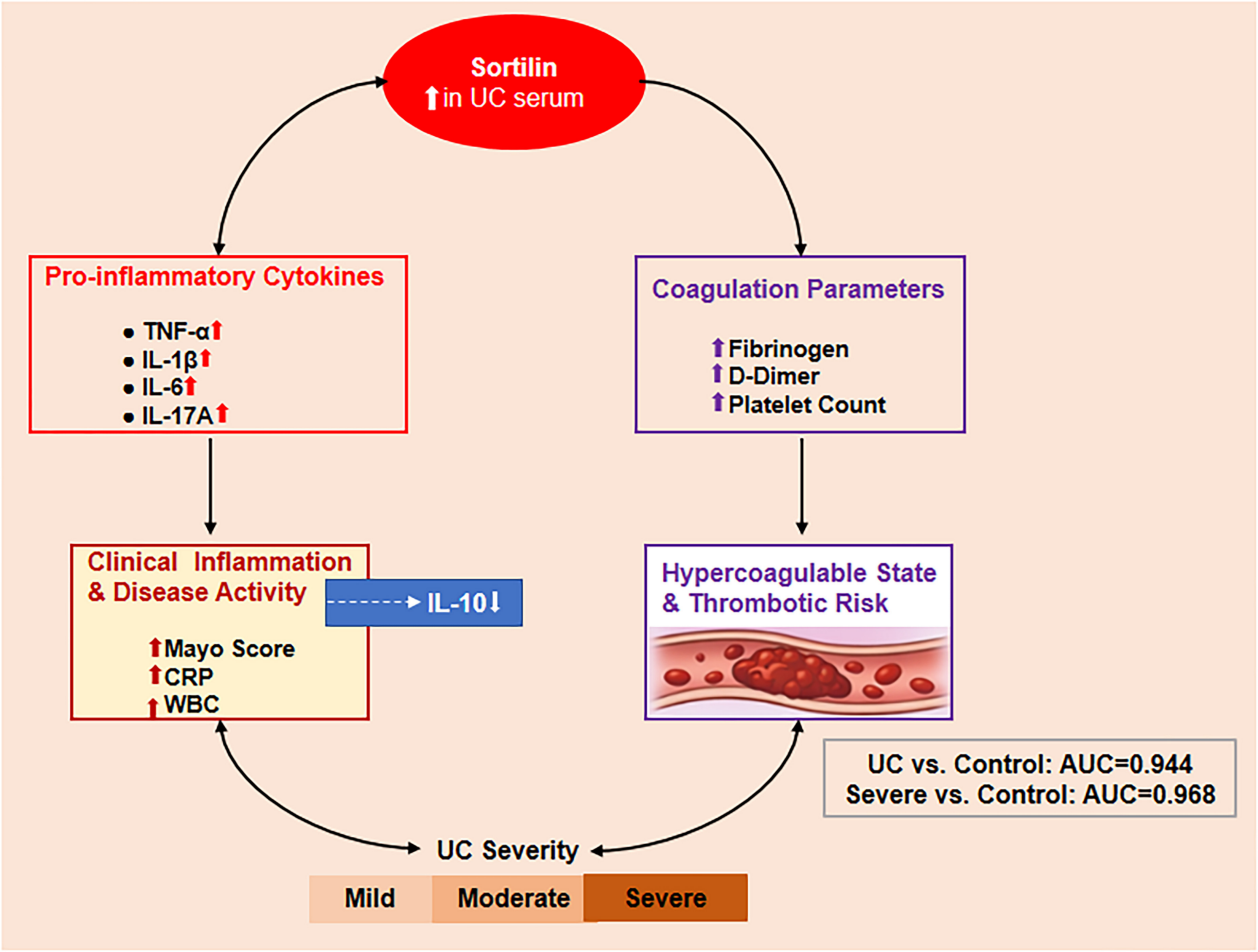
Mechanistic role of elevated serum sortilin in UC. Elevated serum sortilin in UC patients (central red oval) is associated with increased pro-inflammatory cytokines (TNF-α, IL-1β, IL-6, IL-17A; left panel) and decreased anti-inflammatory IL-10 (blue arrow), contributing to enhanced clinical inflammation, including higher Mayo score, CRP, and WBC count. Concurrently, sortilin is positively correlated with coagulation parameters (fibrinogen, D-dimer, and platelet count; right panel), promoting a hypercoagulable state and thrombotic risk. Both the inflammatory and coagulation pathways contribute to UC severity, progressing from mild to severe disease (bottom gradient bar). Correlation coefficients (r) between sortilin and measured parameters are indicated along the arrows. The diagnostic performance of serum sortilin is shown in the inset: UC vs. control (AUC = 0.944) and severe vs. mild UC (AUC = 0.968).

In this study, serum sortilin levels were identified as a novel biomarker that was significantly elevated in UC and closely associated with disease severity, systemic inflammation, and coagulation activation. While serum sortilin shows promise as a biomarker reflecting inflammatory burden and coagulation activation in UC, its diagnostic utility requires further validation in larger, independent cohorts and in comparison with other inflammatory conditions. Although these strong associations suggest a potential mechanistic involvement in UC pathophysiology, causality cannot be established from our cross-sectional study. The correlation of sortilin with key pathogenic cytokines indicates that it may contribute functionally to the UC inflammatory cascade (Figure 7). Future studies should aim to clarify the precise cellular sources and mechanistic roles of sortilin in gut inflammation and validate its potential utility for improving disease monitoring and patient management in UC.

## Data Availability

The datasets presented in this study can be found in online repositories. The names of the repository/repositories and accession number(s) can be found in the article/supplementary material.
